# General hyperconcentration of photonic polarization-time-bin hyperentanglement assisted by nitrogen-vacancy centers coupled to resonators

**DOI:** 10.1038/srep35922

**Published:** 2016-11-02

**Authors:** Fang-Fang Du, Fu-Guo Deng, Gui-Lu Long

**Affiliations:** 1 State Key Laboratory of Low-Dimensional Quantum Physics and Department of Physics, Tsinghua University, Beijing 100084, China; 2 Department of Physics, Applied Optics Beijing Area Major Laboratory, Beijing Normal University, Beijing 100875, China; 3 Tsinghua National Laboratory of Information Science and Technology, Beijing 100084, China; 4 Collaborative Innovation Center of Quantum Matter, Beijing 100084, China

## Abstract

Entanglement concentration protocol (ECP) is used to extract the maximally entangled states from less entangled pure states. Here we present a general hyperconcentration protocol for two-photon systems in partially hyperentangled Bell states that decay with the interrelation between the time-bin and the polarization degrees of freedom (DOFs), resorting to an input-output process with respect to diamond nitrogen-vacancy centers coupled to resonators. We show that the resource can be utilized sufficiently and the success probability is largely improved by iteration of the hyper-ECP process. Besides, our hyper-ECP can be directly extended to concentrate nonlocal partially hyperentangled *N*-photon Greenberger-Horne-Zeilinger states, and the success probability remains unchanged with the growth of the number of photons. Moreover, the time-bin entanglement is a useful DOF and it only requires one path for transmission, which means it not only economizes on a large amount of quantum resources but also relaxes from the path-length dispersion in long-distance quantum communication.

Quantum entanglement is the key important resource for quantum communication, such as quantum teleportation^1^, quantum dense coding^2^,^3^, quantum key distribution^4^,^5^, quantum secret sharing^6^, and quantum secure direct communication^7^,^8^ as entangled photons are generally considered as the ideal information carriers and are used to connect distant quantum nodes in long-distance quantum communication on account of its high-speed transmission and striking low-noise features. Usually entangled photon pairs are produced locally. The photon loss and decoherence caused by the interaction between the photonic quantum system and its environment will inevitably decrease its entanglement during the practical entanglement distribution in long-distance quantum communication.

After passing through a noisy channel, the maximally entangled photon states decay into less entangled pure states or mixed states, leading to the destruction on the fidelity and the security of long-distance quantum communication protocols. In order to depress the decoherence effect on the entangled systems, two interesting quantum techniques, entanglement purification and entanglement concentration, could be exploited to obtain high-fidelity entangled photon systems. In detail, entanglement purification^9^,^10^,^11^,^12^,^13^,^14^ is used to distill a subset of highly entangled states from a set of mixed entangled states, while entanglement concentration^15^,^16^,^17^,^18^ is to extract the maximally entangled states from less entangled pure states. Since Bennett *et al.*^15^ proposed the first entanglement concentration protocol (ECP) for two-photon systems relying on the Schmidt projection method, some good ECPs^16^,^17^,^18^ have been presented.

Hyperentanglement, the entanglement simultaneously in multiple degrees of freedom (DOFs) of a quantum system^19^, has some important applications in quantum communication. It can increase the channel capacity of quantum communication^20^,^21^,^22^,^23^,^24^, achieve the complete Bell-state analysis for the quantum states in the polarization DOF^11^, be used to teleport the unknown quantum state in two DOFs^21^ and complete the hyperentanglement swapping between two photonic quantum systems without entanglement^22^, and help to design the deterministic hyperentanglement purification^11^,^12^,^13^,^14^ which solves the troublesome problem that the parties in quantum repeaters should sacrifice a large amount of quantum resources with conventional entanglement purification protocols (EPPs)^9^,^10^ as the deterministic EPPs^11^,^12^,^13^,^14^ work in a completely deterministic way^25^,^26^,^27^. Recently, some interesting hyperentanglement concentration protocols (hyper-ECPs)^28^,^29^,^30^,^31^,^32^,^33^,^34^,^35^ were proposed. For example, in 2013, Ren *et al.*^28^ proposed the first hyper-ECP for two-photon systems in polarization-spatial less-hyperentangled states with linear optical elements only, including the cases for the nonlocal photonic quantum systems with known and unknown parameters, respectively. More interestingly, they proposed the parameter-splitting method^28^, a fascinating method, to extract the maximally entangled photons when the coefficients of the initial partially entangled state are known, and this method is very efficient and simple in terms of concentrating partially entangled state as it can be achieved with the maximum success probability by performing the protocol only once. In 2014, Li and Ghose^30^ proposed a hyperconcentration scheme for nonlocal *N*-photon hyperentangled Greenberger-Horne-Zeilinger (GHZ) states via linear optics. Sequentially, they^31^ presented two efficient schemes for concentration of nonlocal *N*-photon hyperentanglement based on the cross-Kerr nonlinearity. In 2016, Liu *et al.*^34^ presented a hyper-ECP for the partially hyperentangled *N*-particle GHZ state assisted by a less-entangled *N*-particle GHZ state and three single photons. So far, most of the existing hyper-ECPs^28^,^29^,^30^,^31^,^32^,^33^,^34^ focus on less-hyperentangled states in the polarization and spatial modes DOFs. In 2015, Li and Ghose^35^ presented two hyper-ECPs for two-photon states that are partially entangled in the polarization and time-bin DOFs with linear optics.

The electronic spin associated with a diamond nitrogen vacancy (NV) center is an exceptional solid-state spin qubit system due to optical controllability^36^,^37^,^38^,^39^ and exceeding 10 ms coherence time by using dynamical decoupling techniques^40^. The electron spin of the NV center can be exactly initialized^41^, manipulated^36^,^37^,^38^,^39^,^41^ and read out^42^,^43^. Therefore, the diamond NV center is an attractive platform for quantum information processing due to its long-lived coherence time at room temperature. Many interesting approaches for quantum computation and quantum communication have been proposed based on the NV center in diamond coupled to an optical cavity in theory^24^,^29^,^44^,^45^,^46^ and implemented in experiment^40^,^41^,^47^,^48^,^49^,^50^,^51^,^52^. For example, Ren *et al.*^29^ presented the spatial-polarization photonic hyperentanglement purification and concentration resorting to the nonlinear optics of the NV center embedded in a photonic crystal cavity coupled to a waveguide in 2013. In 2015, Liu and Zhang^24^ presented two interesting schemes for the generation and complete nondestructive analysis of hyperentanglement assisted by nitrogen-vacancy centers in resonators. Jelezko *et al.*^41^ have experimentally demonstrated a conditional controlled quantum gate on electron-nuclear spins of an NV center in 2004. The creation of an entanglement between two distant NV electron spins^40^, a single photon and an NV electron spin^47^, the electron and nearby nuclear spins^48^ have been experimentally demonstrated.

In this paper, we investigate a general hyper-ECP for two-photon systems in an arbitrary partially hyperentangled unknown Bell state that decays with the interrelationship between the time-bin and the polarization DOFs. Our hyper-ECP is achieved by the Schmidt projection method and two parity-check gates that are constructed with the optical property of the NV center coupled to a resonator. By iteration of the hyper-ECP process, the success probability of our hyper-ECP becomes much higher than that in the hyper-ECP with linear optics. At last, we show that our hyper-ECP is suitable for arbitrary partially hyperentangled *N*-photon GHZ states, and the success probability is still unchanged with the growth of the number of photons.

## Results

### The optical property of an NV-cavity platform

**Figure 1:**
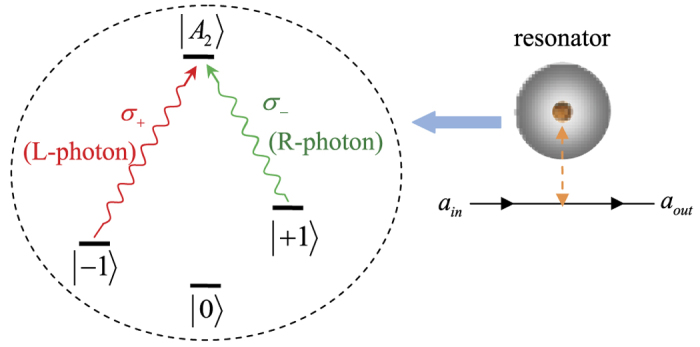
Schematic diagram of a diamond NV center coupled to a resonator. The left ellipse represents the optical transition of the NV-center between the ground states |±1〉 and the excited state |*A*_2_〉.

An NV center in diamond is created by a replaceable nitrogen atom substituting for a carbon atom and an adjacent vacancy in the diamond lattice. The ground state of the NV center is an electronic spin triplet state holding a 2.8 GHz zero-field splitting between the magnetic sublevels |*m*_*s*_ = 0〉 and |*m*_*s*_ = ±1〉 in virtue of the spin-spin interaction^53^. One of the six excited states^54^ 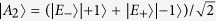 is very robust against the relatively low strain, which is lower than the spin-orbit splitting, and magnetic fields possessing the stable symmetry properties protected by an energy gap retaining the polarization properties of its optical transitions^55^,^56^. Here the electric triple states of the ground *m*_*s*_ = 0 and *m*_*s*_ = ±1 represent |0〉 and |±1〉, respectively. |*E*_±_〉 indicate the orbital states with the angular momentum projections ±1 along the NV axis. In the presence of the small external magnetic field (2π × 200 MHz) which has little effect on the symmetry properties of the |*A*_2_〉 state, the twofold degenerace |*m*_*s*_ = ±1〉 sublevel is split in two levels. The transitions frequency between |±1〉 and |*A*_2_〉 is in the optical regime, i.e., |±1〉 ↔ |*A*_2_〉 are driven by the *σ*_−_ (left − L) and *σ*_+_ (right − R) circularly polarized photon at ~637 nm (shown in Fig. 1), respectively.

The schematic diagram of a diamond NV center coupled to a resonator is shown in Fig. 1. An incident single photon with frequency *ω*_*p*_ enters a single-sided cavity with frequency *ω*_*c*_, which traps a ^-type three-level diamond NV center with frequency difference *ω*_0_ between |−1〉 and |*A*_2_〉. The cavity mode 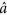 is driven by the input field 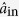. By solving the Heisenberg equations of motion for the annihilation operation 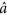 of cavity mode and the lowing operation *σ*_−_ of the NV center^57^,


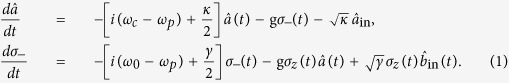


In the weak excitation limit 〈*σ*_*z*_〉 = −1, one can obtain the reflection coefficient for the NV-cavity system^58^,^59^


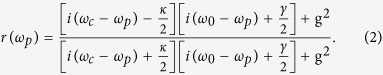


Here the cavity output field 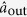 is connected with the input field by the input-output relation 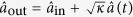. The vacuum input field *b*_*in*_(*t*) has the standard commutation relation 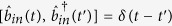. *κ* and *γ* are the decay rates of the cavity and the spontaneous emission rate of the NV center, respectively. g is the coupling rate of the NV-cavity system. In the case of g = 0 in which the NV center is uncoupled from the cavity, Eq. (2) could convert into 
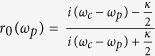
 for an empty cavity.

It is usually not difficult to find that if the photon feels a hot cavity (g ≠ 0), it will get a phase *e*^*iφ*^ after reflection. Otherwise, if the photon feels a cold cavity, it will obtain a phase shift 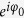. Supposed that the NV center is initially prepared in the state |−1〉, the only possible transition is |−1〉 ↔ |*A*_2_〉. The *L* polarized photon feels a hot microtoroidal resonator (MTR), while *R* polarized photon would sense a bare MTR due to a polarization mismatch, and the corresponding output states of the *L* and *R* photons can be obtained as^45^


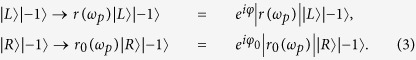


In contrast, if the NV is prepared in the state |+1〉, the input pulse *L (R*) always feel a bare cavity due to the polarization mismatch (the large detuning), and the corresponding output states of the *L* and *R* photons can be obtained as


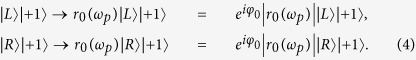


The phase shifts are the function of the frequency detuning (*ω*_*p*_ − *ω*_*c*_) under the resonant condition *ω*_*c*_ = *ω*_0_. By adjusting *ω*_*p*_ = *ω*_*c*_ = *ω*_0_, one can see that the reflection coefficients for the hot cavity *r(ω*_*p*_) and the cold cavity *r*_0_(*ω*_*p*_) can be written as


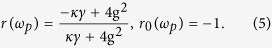


If the condition satisfies 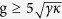, 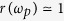 and *r*_0_(*ω*_*p*_) = −1 can be obtained. That is, the spin-selective optical transition rules can be described as


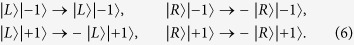


**Figure 2:**
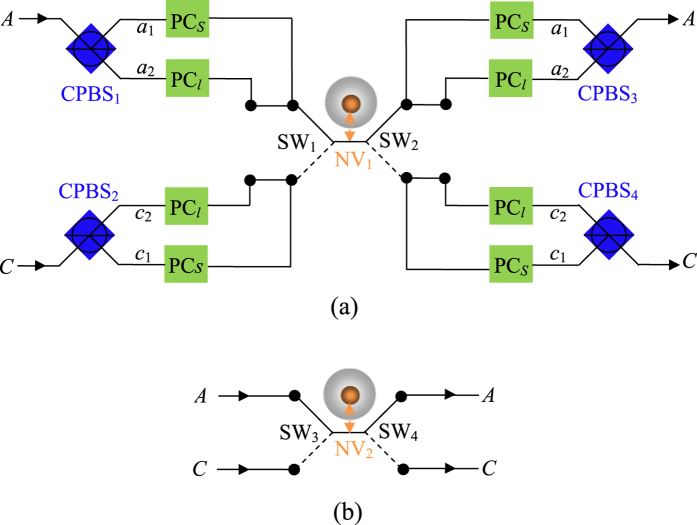
Schematic diagrams of parity-check gates for a two-photon system. (**a**) The time-bin parity-check gate (T-PCG). (**b**) The polarization parity-check gate (P-PCG). NV_1_ (NV_2_) represents a one-side cavity-NV-center system which is used to perform T-PCG (P-PCG) on the photon pair *AC*. CPBS_*i*_ (*i* = 1, 2, …) represents a polarizing beam splitter in the circular basis, which transmits the photon in the right-circular polarization |*R*〉 and reflects the photon in the left-circular polarization |*L*〉, respectively. SW_*j*_(*j* = 1, 2, 3, 4) is an optical switch which makes the wave-packet of a photon successively enter into (or keep away from) the NV center. The PC_*l*_ (PC_*s*_) is a Pockels cell which affects a bit-flip operation on the polarization DOF of the photon at specific times only when the *l(s*)-path component is present.

This input-output property of a cavity-NV-center system can be used to construct the parity-check gates (PCGs) for a two-photon system in both the time-bin (NV_1_ in Fig. 2(a)) and the polarization (NV_2_ in Fig. 2(b)) DOFs.

### Parity-check gates for a two-photon system

A two-photon system *AC* has four Bell states in the time-bin DOF and the four Bell states in the polarization DOF. After passing through a noisy channel, the maximally entangled photon states decay into less entangled pure states denoted as


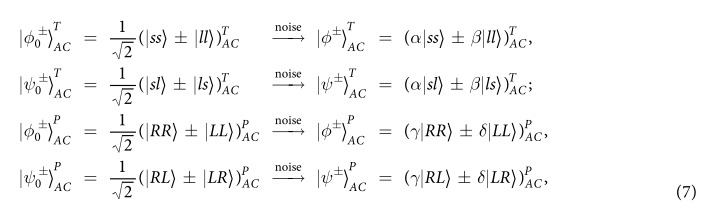


where |*α*|^2^ + |*β*|^2^ = |*γ*|^2^ + |*δ*|^2^ = 1. The Bell states 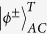 (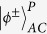) and 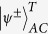 (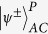) stand for the even-parity and the odd-parity modes in the time-bin (polarization) DOF of the two photon system, respectively. Here the superscripts *T* and *P* represent the time-bin and the polarization DOFs of the two-photon system, respectively. *s* and *l* are the two different time-bins, the early (*s*) and the late (*l*), which possesses the time interval Δ*t* between the two time-bins. *R* and *L* denote the right-circular and left-circular polarizations of photons, respectively.

The parity-check gate (T-PCG) for a two-photon system in the time-bin DOF is constructed with circularly polarizing beam splitters (CPBSs), PC_*s(l*)_, NV_1_, and SWs, shown in Fig. 2(a). CPBS_*i*_ (*i* = 1, 2, …) represents a polarizing beam splitter in the circular basis, which transmits the photon in the right-circular polarization |*R*〉 and reflects the photon in the left-circular polarization |*L*〉, respectively. The PC_*s*_ (PC_*l*_) in the spatial-mode *a*_1_ and *c*_1_ (*a*_2_ and *c*_2_) is a Pockels cell (PC)^60^ which performs a bit-flip operation (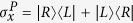) on the polarization DOF of the photon *A (C*) at the specific time only when the *s(l*)-path component appears. That is,


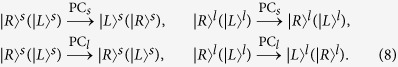


It is worth pointing out that our PC_*s*_ (PC_*l*_) is consisted of two half-wave plates (HWPs) (indicating the mutual transformation between the circular polarization and linear polarization) and the PC′_*s*_ (PC′_*l*_) shown in ref. 60. Taken the transformation 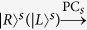 |*L*〉^*s*^(|*R*〉^*s*^) as an example, it can be accomplished with the processes 
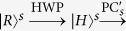
 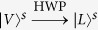. SW_*j*_ (*j* = 1, 2) is an optical switch which makes the wave-packet of the photons *A* and *C* successively enter into (or keep away from) the NV_1_ center. Suppose that the initial state of the two-photon system *AC* is a Bell state 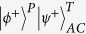 and that of the auxiliary NV_*i*_ is 
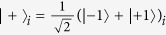
 with *i* = 1, 2, Alice lets the photon *A (C*) pass through CPBS_1_ (CPBS_2_), PC_*s(l*)_, SW_1_, NV_1_, SW_2_, PC_*s(l*)_, and CPBS_3_ (CPBS_4_) in sequence, and the state 
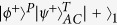
 of the complicated system composed of NV_1_ and the two-photon system *AC* evolves into


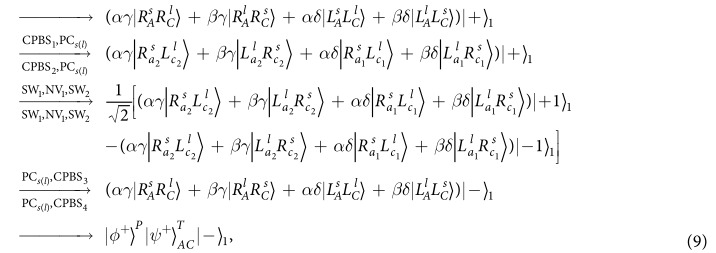


where 
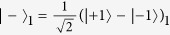
. Similarly, after interaction, the evolutions of the other states of the system can be described as follows:


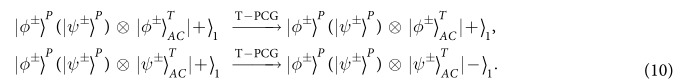


It is quite clear that the polarization states of the two photons *AC* have not be affected. By measuring the auxiliary NV_1_ in the orthogonal basis {|+〉,|−〉}, one can distinguish the even-parity time-bin Bell states from the odd-parity ones. That is, if the auxiliary NV_1_ is projected into the state |+〉_1_, the time-bin state is the even-parity one; otherwise, it is the odd-parity one.

The principle of our polarization parity-check gate (P-PCG) is shown in Fig. 2(b) and it is used to distinguish the parity of the hyperentangled Bell states in polarization DOF. Similar to our T-PCG, if one lets the photons *A* and *C* pass through the quantum circuit shown in Fig. 2(b) in sequence, the rule for the evolutions of quantum states of the complicated system composed of two photons *AC* and the auxiliary NV_2_ is


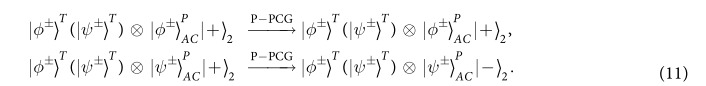


By measuring the auxiliary NV_2_ in the orthogonal basis {|+〉,|−〉}, one can distinguish the hyperentangled Bell states with the even-parity mode from those with the odd-parity mode in the polarization DOF without influencing the states of the photons in the time-bin DOF. In detail, if the auxiliary NV_2_ is projected into the state |+〉_2_, the polarization state is the even-parity mode; otherwise, it is the odd-parity one.

### High-efficiency hyper-ECP for arbitrary two-photon systems

In a long-distance quantum communication, the maximally hyperentangled Bell state 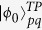 in both the time-bin and polarization DOFs may decay to the partially hyperentangled Bell-type state by the independent decoherence of the entanglement in two DOFs^35^. Also, the maximally hyperentangled Bell state 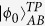 will decay to an arbitrary partially hyperentangled Bell state 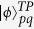 if the interrelation between the time-bin and the polarization DOFs is taken into account. Here


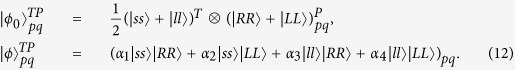


**Figure 3:**
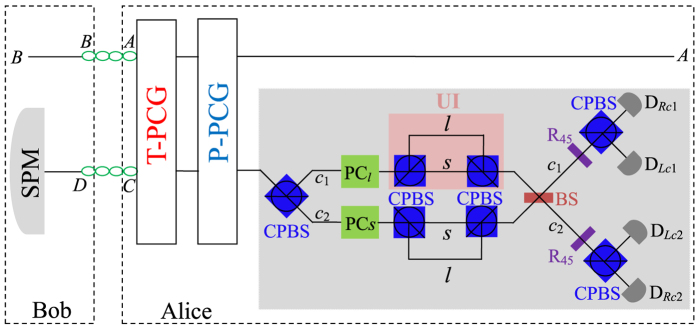
Schematic diagram of our hyper-ECP for two-photon systems in an arbitrary partially hyperentangled Bell state, resorting to T-PCG and P-PCG. The length difference between the *l* and *s* paths in the unbalanced interferometer (UI) is designed to cancel the time interval between the two time-bins. R_45_ represents a half-wave plate which is used to perform a Hadamard operation on the polarization DOF of the photons. BS represents a 50:50 beam splitter. 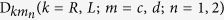 represents a single-photon detector. SPM represents a single-photon measurement device which is similar to the one performed by Alice in the grey rectangle by replacing *d* with *c*.

Here the subscripts *p* and *q* present the photons held by two distant parties, Alice and Bob, respectively. The four unknown parameters *α*_1_, *α*_2_, *α*_3_, and *α*_4_ satisfy the normalization condition |*α*_1_|^2^ + |*α*_2_|^2^ + |*α*_3_|^2^ + |*α*_4_|^2^ = 1, and three unknown parameters are independent. The principle of our general hyper-ECP for two-photon systems in both the polarization and the time-bin DOFs is shown in Fig. 3. It includes two steps which can be described in detail as follows.

(1) **The first step of our hyper-ECP for the two-photon systems.** To realize the first step of our hyper-ECP with the Schmidt projection method, a pair of the two-photon systems *AB* and *CD* from a set of the two-photon systems in the state 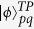 is required for each process in hyper-ECP. That is, the two photons *A*, and *C* belong to Alice, and the other two photons *B* and *D* belong to Bob.

The principle of the first step of our hyper-ECP for the photon pairs *AB* and *CD* is shown in Fig. 3. The initial state of the four-photon system *ABCD* can be written as 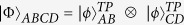. Alice performs the T-PCG and P-PCG on the photon pair *AC*. The outcomes can be divided into four groups, and they are discussed in detail as follows.

(1.1) If the outcomes of the T-PCG and P-PCG are in an even-parity time-bin mode and an odd-parity polarization mode, respectively, the four-photon system is projected into the state |Φ_1*eo*_〉_*ABCD*_ with the probability of *p*_1*eo*_ = 2(|*α*_1_*α*_2_|^2^ + |*α*_3_*α*_4_|^2^). Here


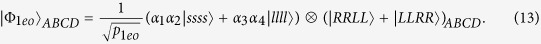


Subsequently, Alice (Bob) performs the single-photon measurement (SPM) on the photon *C (D*). The SPM setup is composed of linear optical elements, shown in Fig. 3. The effect of the unbalanced interferometer (UI) can be described as 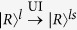 and 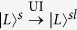. Here the length difference between the two arms *s* and *l* is set exactly to *c*Δ*t*, where *c* is the speed of the photons. After passing through two PCs and two UIs, the state |Φ_1*eo*_〉_*ABCD*_ is transformed into the state |Φ′_1*eo*_〉_*ABCD*_. Here


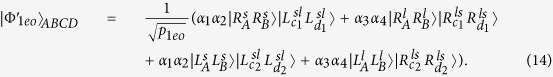


Obviously, both the two photons *C* and *D* will arrive at their SPMs at the same time, respectively, i.e., in the middle time slot. However, there are two potential spatial modes for each photon, the up mode *c*_1_(*d*_1_) and the down mode *c*_2_(*d*_2_), which makes the two photons *C* and *D* be measured in both the polarization DOF and the spatial mode DOF. The effect of a 50:50 beam splitter (BS) can be described as 
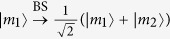
 and 
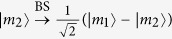
 (*m* = *c*, *d*). R_45_ is used to perform a Hadamard operation on the polarization DOF of the photons, that is, 
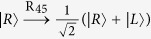
 and 
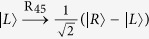
. At last, the two photons *C* and *D* are detected by the single-photon detectors. The relationship between the measurement results of the detectors and the shared states |*ϕ*_1*eo*_〉_*AB*_ is shown in Table 1. If two detectors (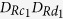, 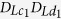, 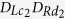, or 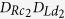) are clicked, the two-photon system *AB* is projected into the state 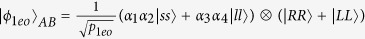, which is the partially hyperentangled Bell-type state with the polarization DOF in a maximally entangled Bell state. For the other three cases of the two clicked detectors, a phase flip operation 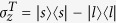, 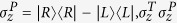 in sequence on the photon *B* is required to obtain the partially hyperentangled Bell-type state |*ϕ*_1*eo*_〉_*AB*_.

(1.2) If the outcomes of the T-PCG and P-PCG are in an odd-parity time-bin mode and an even-parity polarization mode, respectively, the four-photon system is projected into the state |Φ_1*oe*_〉_*ABCD*_ with the probability of *p*_1*oe*_ = 2(|*α*_1_*α*_3_|^2^ + |*α*_2_*α*_4_|^2^). Here


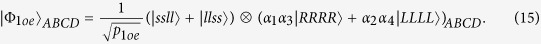


Similar to the discussion above, after two parters perform the SPMs on the photons *C* and *D*, two-photon system *AB* is projected into the hyperentangled Bell state 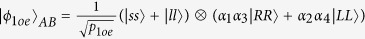 with the time-bin DOF in a maximally entangled Bell state by assisting the conditional phase flip operation *Y* (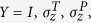 or 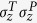) on photon *B* depending on the measurement results of the detectors shown in Table 1.

(1.3) If the outcomes of the T-PCG and P-PCG are in an odd-parity time-bin mode and an odd-parity polarization mode, respectively, the four-photon system is projected into the state |Φ_1*oo*_〉_*ABCD*_ with the probability of *p*_1*oo*_ = 2(|*α*_1_*α*_4_|^2^ + |*α*_2_*α*_3_|^2^). Here


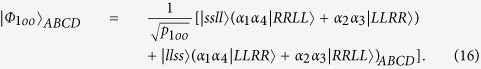


Alice and Bob make the photons *A*, *B*, *C*, and *D* pass through the PC_*l*_, PC_*l*_, PC_*s*_, and PC_*s*_, respectively, which realize the polarization bit-flip operations on the time-bin modes *l*_*A*_, *l*_*B*_, *s*_*C*_, and *s*_*D*_, respectively. Then the state |Φ_1*oo*_〉_*ABCD*_ is transformed into 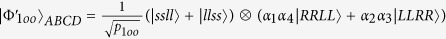. Similar to the case (1.2), after replacing *α*_1_*α*_3_ and *α*_2_*α*_4_ with *α*_1_*α*_4_ and *α*_2_*α*_3_, respectively, the state of the two-photon system *AB* is transformed into 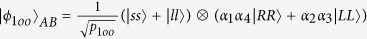.

(1.4) If the outcomes of the T-PCG and P-PCG are in an even-parity time-bin mode and an even-parity polarization mode, respectively, the four-photon system is projected into the state |Φ_1*ee*_〉_*ABCD*_ with the probability of 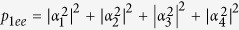. Here


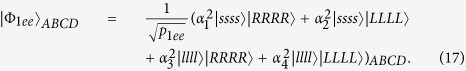


Alice and Bob perform the SPMs on the photons *C* and *D*, respectively, and then the state |Φ_1*ee*_〉_*ABCD*_ collapses to 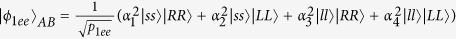, which is the partially hyperentangled Bell state with less entanglement than the state 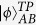.

(2) **The second step of our hyper-ECP for two-photon systems.** In this step, another two photon pairs *A*′*B*′ and *C*′*D*′ in the partially hyperentangled Bell state 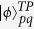 are required, which are identical to the two photon pairs *AB* and *CD*. Here the two photons *A*′*C*′ belong to Alice, and the two photons *B*′*D*′ belong to Bob. Alice and Bob perform the same operations on the photon pairs *A*′*B*′ and *C*′*D*′ as those on the photon pairs *AB* and *CD*, and the same results can be obtained. That is, the four cases |*ϕ*_1*eo*_〉_*A*′*B*′_, |*ϕ*_1*oe*_〉_*A*′*B*′_, |*ϕ*_1*oo*_〉_*A*′*B*′_, and |*ϕ*_1*ee*_〉_*A*′*B*′_ are obtained with the probabilities *p*_1*eo*_, *p*_1*oe*_, *p*_1*oo*_, and *p*_1*ee*_, respectively, by replacing the four photons *ABCD* with *A*′*B*′*C*′*D*′. Alice and Bob will distill a maximally hyperentangled Bell state 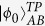 from the partially hyperentangled Bell-type states obtained by the above three cases of the first step. The principle of this step is the same as the first one shown in Fig. 3 by replacing the photons *ABCD* with *ABA*′*B*′. That is, Alice performs the T-PCG and P-PCG on the photon pairs *AA*′.

(2.1) For the case in (1.1), the state of the four-photon system *ABA*′*B*′ is |Ψ_1_〉_*ABA*′*B*′_ = |*ϕ*_1*eo*_〉_*AB*_ ⊗ |*ϕ*_1*eo*_〉_*A*′*B*′_. Alice picks up the case when the outcome of the T-PCG is in an odd-parity time-bin mode, and a maximally hyperentangled Bell state can be obtained whether the outcome of the P-PCG is in an odd-parity polarization mode or in an even-parity polarization mode. That is, the four-photon system is projected into the states |Ψ_1*oe*_〉_*ABA*′*B*′_ and |Ψ_1*oo*_〉_*ABA*′*B*′_ with the same probability *p*_1_ = *p*_1*oy*_/*p*_1*eo*_ (*p*_1*oy*_ = 4|*α*_1_*α*_2_*α*_3_*α*_4_|^2^), respectively. Here


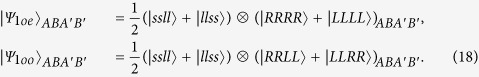


After performing the SPMs on the photons *A*′ and *B*′ and assisting the conditional phase-flip operation on the photon *B*, the two-photon system *AB* is projected into the maximally hyperentangled Bell state 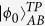 with the probability 2*p*_1_.

If the outcome of the T-PCG is in an even-parity time-bin mode, the four-photon system is projected into the states |Ψ_1*eo*_〉_*ABA*′*B*′_ and |Ψ_1*ee*_〉_*ABA*′*B*′_ with the same probability *p*′_1_/*p*_1*ey*_ = *p*_1*eo*_, respectively. Here


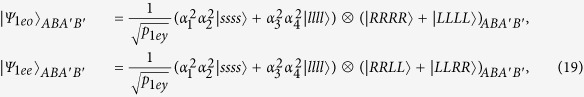


where 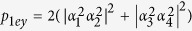. After measuring the photons *A*′ and *B*′, the two-photon system *AB* is projected into the partially hyperentangled Bell-type state 
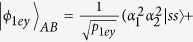
 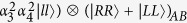 with the probability 2*p*′_1_, which can be used in the second step in the second round of the hyper-ECP process.

(2.2) For the case in (1.2), the state of the four-photon system *ABA*′*B*′ is |Ψ_2_〉_*ABA*′*B*′_ = |*φ*_1*oe*_〉_*AB*_ ⊗ |*φ*_1*oe*_〉_*A*′*B*′_. Likewise, Alice and Bob pick up the case when the outcome of the P-PCG is in an odd-parity polarization mode, and a maximally hyperentangled Bell state 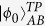 with the probability 2*p*_2_(*p*_2_ = *p*_1*yo*_/*p*_1*eo*_, *p*_1*yo*_ = *p*_1*oy*_) can be obtained whether the outcome of the T-PCG is in an odd-parity time-bin mode or in an even-parity polarization mode. If the outcome of the P-PCG is in an even-parity time-bin mode, the two-photon system *AB* is projected into the partially hyperentangled Bell-type state 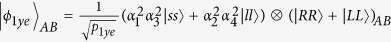 with the same probability 2*p′_2_* 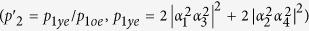.

(2.3) For the case in (1.3), the same operations are performed on the state |Ψ_3_〉_*ABA*′*B*′_ = |*φ*_1*oo*_〉_*AB*_⊗|*φ*_1*oo*_〉_*A*′*B*′_ as those on the state |Ψ_2_〉_*ABA*′*B*′_ in case (2.2). The maximally hyperentangled Bell state 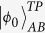 can be obtained with the probability 2*p*_3_(*p*_3_ = *p*_1*xo*_/*p*_1*oo*,_ *p*_1*xo*_ = 4|*α*_1_*α*_2_*α*_3_*α*_4_|^2^) and the state 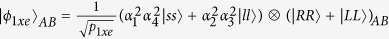 can be obtained with the probability 2*p*′_3_(*p*′_3_ = *p*_1*xe*_/*p*_1*oo*_, 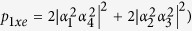.

**Figure 4:**
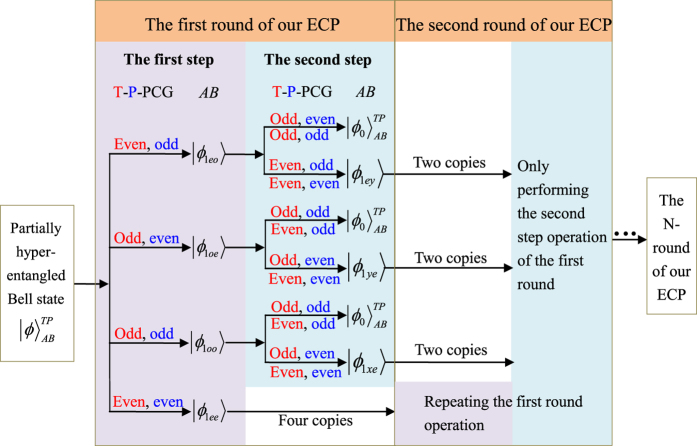


In the hyper-ECP for the two-photon systems in the partially hyperentangled Bell state with linear optics in ref. 35, only one of the three cases (1.1), (1.2), and (1.3) can be preserved, and only one of the two time-bin (polarization) parity cases (the even parity or odd parity of the case (2.1), (2.2), or (2.3)) is preserved in the second step. These six cases can all be preserved in our hyper-ECP with the NV center, so the success probability *P*_1_ of the first round of the hyper-ECP process is almost five times larger than that in the hyper-ECP with linear optical elements. The success probability in ref. 35 is *P* = *m*, *m* ∈ (*p*_1_, *p*_2_, *p*_3_). After the first round of our hyper-ECP, the total success probability of the maximally hyperentangled Bell state 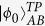 is *P*_1_ = 2(*p*_1_ + *p*_2_ + *p*_3_). The left of Fig. 4 shows the procedure of the first round of our ECP for two-photon systems in an arbitrary partially hyperentangled Bell state with T-P-PCG in detail.

Now, let us discuss the second round of our ECP for two-photon systems in an arbitrary partially hyperentangled Bell state with T-P-PCG shown in the right of Fig. 4. For the partially hyperentangled Bell state |*ϕ*_1*ee*_〉_*AB*_ preserved in the case (1.4), requiring four copies of the two-photon systems to complete the two steps in the second round of the hyper-ECP process. While for the hyperentangled Bell-type states |*ϕ*_1*ey*_〉_*AB*_, |*ϕ*_1*ye*_〉_*AB*_, and |*ϕ*_1*xe*_〉_*AB*_ preserved in the second step, two copies of the photon systems in each state are required to complete only the second step in the second round of the hyper-ECP process. After the second round of the hyper-ECP, the success probability of the maximally hyperentangled Bell state 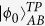 is


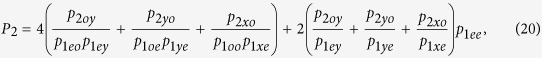


where 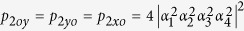. Again, the partially hyperentangled Bell states preserved in the *n*-th round of the hyper-ECP process can be used to distill the maximally hyperentangled Bell state in the (*n* + 1)-th round. The success probability of the hyperentanglement concentration process in *n*-th (*n* > 2) round can be described as follows


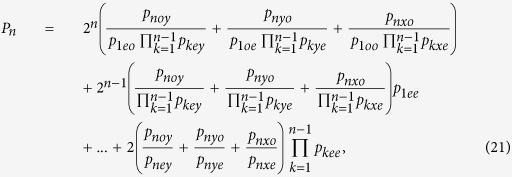


where


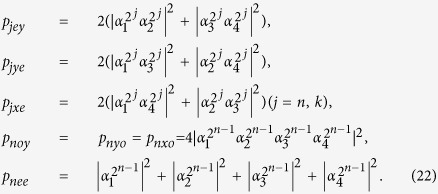


After the *n*-th round iteration of the hyper-ECP process, the total success probability is 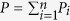. The success probabilities equal to *P*_1_ = 0.225, *P*_2_ = 0.312, and *P*_5_ = 0.338 when |*α*_1_| = 0.3, |*α*_3_| = 0.4, and |*α*_2_| = |*α*_4_|. Further, they can be increased to *P*_1_ = 0.353, *P*_2_ = 0.536, and *P*_5_ = 0.665 with |*α*_1_| = |*α*_3_| = 0.45, and |*α*_2_| = |*α*_4_|. If the conditions become |*α*_1_| = |*α*_3_| = 0.5, |*α*_2_| = 0.51, and 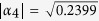, the success probabilities raise to *P*_1_ = 0.375, *P*_2_ = 0.585, and *P*_5_ = 0.762. Obviously, with the iteration of our hyper-ECP process, the total success probability *P* will be increased largely.

## Discussion

It is clear that our hyper-ECP can be generalized to distill a maximally hyperentangled *N*-photon GHZ state from an arbitrary partially hyperentangled GHZ-class state that decays with the interrelation between the time-bin and polarization DOFs. Suppose an arbitrary partially hyperentangled *N*-photon GHZ state is described as


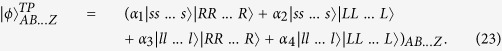


The subscripts *A*, *B*, …, and *Z* represent the photons that are kept by the remote users, called Alice, Bob, …, and Zach, respectively. As there are also three independent parameters in the state 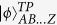, which is similar to the state 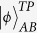, our hyper-ECP can be directly extended to distill maximally hyperentangled *N*-photon GHZ states 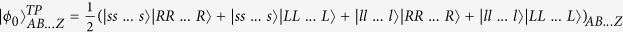 with the operations performed by Alice only with the T-PCG and P-PCG on her own photon pairs. The success probability of our hyper-ECP for *N*-photon systems in an arbitrary partially hyperentangled GHZ state is the same as the one for two-photon systems in an arbitrary partially hyperentangled Bell state. That is because that only Alice is required to perform the concentration operations, and the remaining *N* − 1 parties do nothing, which can be viewed as Bob with a complicate system in essence (In detail, |*ss *... *s*〉_*AB*...*Z*_ ⇒ |*s*_*A*_*s*_*B*_〉,|*ll *... *l*〉_*AB*...*Z*_ ⇒ |*l*_*A*_*l*_*B*_〉, |*RR *... *R*〉_*AB*...*Z*_ ⇒ |*R*_*A*_*R*_*B*_〉, and |*LL *... *L*〉_*AB*...*Z*_ ⇒ |*L*_*A*_*L*_*B*_〉). Therefore, the success probability remains unchanged with the growth of the number of photons. When the number of photons to be concentrated is large, our scheme may be more efficient and more practical.

The polarization DOF and the spatial mode DOF are the two most popular DOFs of the photon as they are easy to manipulate and measure with linear optical elements. However, using the spatial mode of each photon to carry information requires two paths during the transmission, which may introduce the path-length dispersion in long-distance multi-photon communication. The time-bin states, also as a conventional classical DOF, can be simply discriminated by the time of arrival. The time-bin DOF is very helpful for quantum communication as it can be used to accomplish the faithful qubit transmission without ancillary qubits^61^, the deterministic two-photon entanglement purification^62^, the arbitrary multi-photon entanglement sharing^63^, and the complete error correction for nonlocal spatial-polarization hyperentangled photon pairs^64^.

In summary, we have proposed a general hyper-ECP for improving the entanglement of the two-photon systems in an arbitrary partially hyperentangled Bell state that decays with the interrelationship between the time-bin and the polarization DOFs, resorting to the T-PCG and P-PCG that are constructed by the optical property of NV-cavity systems. Our hyper-ECP is different from the hyper-ECP^35^ with unknown parameters, which is focused on the partially hyperentangled pure states accompanied by the independent decoherence in two DOFs. We show that the resource can be utilized sufficiently and the success probability is largely improved by iteration of the hyper-ECP process. The success probability of the first round of our hyper-ECP is almost five times than that in the hyper-ECP^35^ with linear optical elements. In addition, our hyper-ECP can be straightforwardly generalized for arbitrary partially hyperentangled *N*-photon GHZ states, especially for the case with the interrelation between the two DOFs of multi-photon systems, and the success probability remains unchanged with the growth of the number of photons. Besides hyper-ECP, the basic parity-check gate elements, including P-PCG and T-PCG can also be used to construct the high-efficiency hyperentanglement purification protocol for obtaining high-fidelity hyperentangled states from mixed hyperentangled states. Moreover, the time-bin entanglement is a useful DOF and it only requires one path during the transmission process, which means that it not only economizes on a large amount of quantum resources but also relaxes the path-length dispersion in long-distance quantum communication.

## Methods

### Average fidelities and efficiencies of the parity-check gates

In this part, we give a brief discussion about the experimental implementation of our scheme. The NV center in diamond has attracted much attention with millisecond coherence time^37^, and its ground state spin coherence time can be extended to 480 *μ*s with Gaussian decay using a Hahn echo sequence^38^, which may be made further efforts extend much longer by coupling with an optical cavity. The individual diamond NV-center has reached nanosecond occupancy time^39^, and its spin states can be read out nondestructively with spin-dependent photoluminescence. However, it is known that only very little of the total NV spontaneous optical emission is the direct transitions between the ground and the excited states^42^,^53^, and this weak zero phonon line (ZPL) emission presents an experimental challenge to our proposals. In 2009, Barclay *et al.*^49^ showed that it is possible to enhance the ZPL emission rate by 47% if the *Q* of the microdisk can be increased to 2.5 × 10^4^. In recent years, the ZPL emission rate has been enhanced from 3% to 70%^65^,^66^. When the NV center is coupled to the resonator, the spontaneous emission into the ZPL can be largely enhanced, and the interaction of the NV center and photons is also strengthened.

Generally speaking, the reflection rule may be not perfect in experiment. The main factors that reduce the efficiency and fidelity of our scheme are the cavity field decay rate *γ*, cavity side leakage rate *κ* and the coupling strength *g* in the coupled reflection coefficients *r(ω*_*p*_) (*r*_0_(*ω*_*p*_) = −1). Defining the efficiency as the yield of the photons, that is, *η* = *η*_*output*_/*η*_*input*_. Here *η*_*input*_ is the number of the input photon, whereas *η*_*output*_ is the number of the output photon. The fidelity is defined as *F* = |〈*ψ*_*ideal*_|*ψ*_*real*_〉|^2^, i.e., the overlap of the output states of the system in the ideal case |*ψ*_*ideal*_〉 and the realistic case |*ψ*_*real*_〉. The fidelities of the T-PCG and P-PCG in both the even-parity mode and the odd-parity mode are


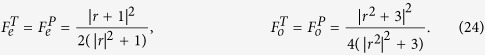


Apparently, the fidelity of the even (odd)-parity mode of the T-PCG equals to the one of the P-PCG. The even (odd)-parity mode in the time-bin DOF can not be directly distinguished, which is different from the ones in the polarization and spatial DOFs. Therefore, the effect of the T-PCG first transfers the even (odd)-parity mode in the time-bin DOF into the same parity mode in the polarization DOF, and then the parity measurement results of the polarization DOF feedback to the ones of the time-bin DOF in essence.

**Figure 5:**
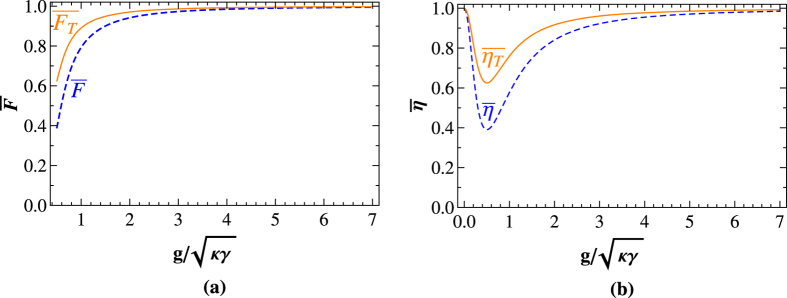
(**a**) The average fidelities 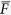 and 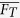 of our T-P-PCG and T-PCG vs the ratio of 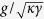 for the case 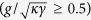, respectively; (**b**) The average efficiencies 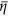 and 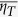 of our T-P-PCG and T-PCG vs the ratio of 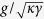 with *ω*_*c*_ = *ω*_*p*_ = *ω*_0_, respectively.

Our hyper-ECP only requires Alice to perform the local parity-check (T-PCG and P-PCG) operations on her own photon pair. Let us define the average fidelity of the T-P-PCG as 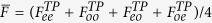, where 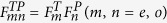 represents the fidelity of the two-photon system paralleling in two DOFs (either the even-parity mode or the odd-parity mode). The average fidelities and efficiencies of our proposal depended mainly on the effect of 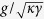, which are shown in Fig. 5(a,b), respectively. For the diamond NV center in the MTR with whispering-gallery microresonator mode system, the research^45^ shows that *r(ω*_*p*_) ~ 0.95 when 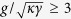 with *ω*_*c*_ = *ω*_*p*_ = *ω*_0_; when 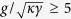 with *ω*_*c*_ = *ω*_*p*_ = *ω*_0_, provided that there is a MTR with 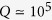 (corresponding to *κ* ~ 1 GHz) or 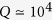 (corresponding to *κ*~10 GHz) according to the experimental results^49^, the coupling strength should be on the order of hundreds of megahertz in order to reach *r(ω*_*p*_)~1. Our T-P-PCG and T-PCG with fidelities and efficiencies greater than 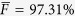, 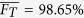, 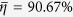, and 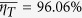 can be achieved when 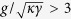, which makes our hyper-ECP easier to be realized. It is not difficult to find that higher fidelity and efficiency can be acquired in the condition of the stronger coupling strength and the lower cavity decay rate.

## Additional Information

**How to cite this article**: Du, F.-F. *et al.* General hyperconcentration of photonic polarization-time-bin hyperentanglement assisted by nitrogen-vacancy centers coupled to resonators. *Sci. Rep.* **6**, 35922; doi: 10.1038/srep35922 (2016).

**Publisher’s note:** Springer Nature remains neutral with regard to jurisdictional claims in published maps and institutional affiliations.

## Figures and Tables

**Table 1:** The relation between the final state of *AB* and the measurement results.

|*φ*_1*eo*_〉_*AB*_	Measurement results
(*α*_1_*α*_2_|*ss*〉 + *α*_3_*α*_4_|*ll*〉) ⊗ (|*RR*〉 + |*LL*〉)	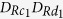 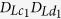 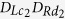 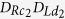
(*α*_1_*α*_2_|*ss*〉 − *α*_3_*α*_4_|*ll*〉) ⊗ (|*RR*〉 + |*LL*〉)	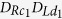 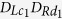 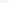 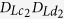 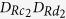
(*α*_1_*α*_2_|*ss*〉 + *α*_3_*α*_4_|*ll*〉) ⊗ (|*RR*〉 − |*LL*〉)	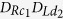 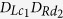 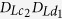 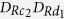
(*α*_1_*α*_2_|*ss*〉 − *α*_3_*α*_4_|*ll*〉) ⊗ (|*RR*〉 − |*LL*〉)	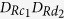 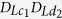 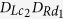 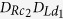
